# Deep transcranial magnetic stimulation for schizophrenia: a systematic review

**DOI:** 10.3389/fpsyt.2024.1390913

**Published:** 2024-05-31

**Authors:** Yu Mo, Zhan-Ming Shi, Xin-Hu Yang, Xian-Jun Lan, Can-Jin Deng, Xing-Bing Huang, Xiao-Lin Tan, Saxby Pridmore, Gabor S. Ungvari, Yu-Tao Xiang, Wei Zheng

**Affiliations:** ^1^ Department of Psychiatry, The Brain Hospital of Guangxi Zhuang Autonomous Region, LiuZhou, China; ^2^ Department of Psychiatry, Chongqing Jiangbei Second Hospital, Chongqing, China; ^3^ Department of Psychiatry, The Affiliated Brain Hospital of Guangzhou Medical University, Guangzhou, China; ^4^ Key Laboratory of Neurogenetics and Channelopathies of Guangdong Province and the Ministry of Education of China, Guangzhou Medical University, Guangzhou, China; ^5^ Department of Psychiatry, Chongqing mental health center, Chongqing, China; ^6^ Discipline of Psychiatry, University of Tasmania, Hobart, TAS, Australia; ^7^ Section of Psychiatry, University of Notre Dame Australia, Fremantle, WA, Australia; ^8^ Division of Psychiatry, School of Medicine, University of Western Australia, Perth, WA, Australia; ^9^ Unit of Psychiatry, Department of Public Health and Medicinal Administration, & Institute of Translational Medicine, Faculty of Health Sciences, University of Macau, Macao, Macao SAR, China; ^10^ Centre for Cognitive and Brain Sciences, University of Macau, Macao, Macao SAR, China

**Keywords:** deep transcranial magnetic stimulation, schizophrenia, psychopathology, executive function, systematic review

## Abstract

**Background:**

The efficacy and safety of deep transcranial magnetic stimulation (dTMS) as an intervention for schizophrenia remain unclear. This systematic review examined the efficacy and safety of dTMS for schizophrenia.

**Methods:**

A systematic search of Chinese (WanFang and Chinese Journal Net) and English databases (PubMed, EMBASE, PsycINFO, and Cochrane Library) were conducted.

**Results:**

Three randomized clinical trials (RCTs) comprising 80 patients were included in the analyses. Active dTMS was comparable to the sham treatment in improving total psychopathology, positive symptoms, negative symptoms, and auditory hallucinations measured by the Positive and Negative Syndrome Scale (PANSS), the Scale for the Assessment of Positive Symptoms (SAPS), the Scale for the Assessment of Negative Symptoms (SANS), and the Auditory Hallucinations Rating Scale (AHRS), respectively. Only one RCT reported the effects on neurocognitive function measured by the Cambridge Neuropsychological Test Automated Battery (CANTAB), suggesting that dTMS may only improve one Stockings of Cambridge measure (i.e., subsequent times for five move problems). All three studies reported overall discontinuation rates, which ranged from 16.7% to 44.4%. Adverse events were reported in only one RCT, the most common being tingling/twitching (30.0%, 3/10), head/facial discomfort (30.0%, 3/10), and back pain (20.0%, 2/10).

**Conclusion:**

This systematic review suggests that dTMS does not reduce psychotic symptoms in schizophrenia, but it shows potential for improving executive functions. Future RCTs with larger sample sizes focusing on the effects of dTMS on psychotic symptoms and neurocognitive function in schizophrenia are warranted to further explore these findings.

## Introduction

Schizophrenia is a psychiatric disorder characterized by psychotic symptoms and impaired cognition, emotional life and insight (1, 2). As a chronic illness, schizophrenia imposes a substantial burden on sufferers, their families, and society due to its high rate of disability (3). However, patients and their families often face barriers in seeking treatment, such as limited insight, real or perceived stigma, and a pessimistic prognosis (4, 5). Effective treatment can enhance engagement between patients and their families, improve quality of life, and contribute to the reintegration of patients into society, thereby reducing the incidence of destructive behaviors such as violence and aggression (6).

Psychopharmacological treatment has been the primary approach in treating schizophrenia since the 1950s. However, a significant proportion of patients has poor treatment response or intolerable drug side effect (7), and up to 70% of patients are resistant to first-line antipsychotic treatments (8). Consequently, in addition to electroconvulsive therapy (ECT) (9, 10), there has been growing interest in exploring adjunctive, non-invasive brain stimulation (NIBS) techniques such as transcranial magnetic stimulation (TMS) (11), magnetic seizure therapy (MST) (12), transcranial direct current stimulation (tDCS) (13), and transcranial alternating current stimulation (tACS) (14). There is preliminary evidence that NIBS may improve psychiatric symptoms (15) and neurocognitive function (16) in patients with schizophrenia. However, these techniques also have limitations. For instance, ECT can negatively affect memory and neurocognitive function (17), and the effectiveness and safety of MST for schizophrenia remain uncertain (18).

A promising new development is deep transcranial magnetic stimulation (dTMS), which addresses the limitations of traditional TMS. Traditional TMS is limited in its ability to accurately stimulate localized targets, as in 27% to 32% of patients the intended stimulation site is not reached (19). When compared to traditional TMS using a 8 coil, dTMS with a specialized (H) coil is more precise and allows for deeper stimulation through brief magnetic pulses that induce targeted neuronal depolarization in the cerebral cortex (20). As a novel technique, dTMS enables non-invasive stimulation of deep layers of the prefrontal cortex. Several studies have found a method of modulating insula function through targeted neurostimulation, which can be achieved using dTMS (21, 22). This technique has demonstrated efficacy in various neurological and psychiatric disorders, including depression (23), obsessive-compulsive disorder (24), and substance use disorders (25). dTMS does not require hospitalization or anesthesia and has minimal side effects (26). It has several further advantages, including deeper stimulation, broader range, reduced damage to the surface cortex, effective reduction of static electric field interference, and slower decay rate (27).

However, the therapeutic effect of dTMS in schizophrenia is still inconsistent. An open-label study found that dTMS significantly improved negative symptoms in schizophrenia (28), but recent randomized controlled trials (RCTs) (29–31) concluded that dTMS did not improve psychotic symptoms in schizophrenia. For example, Rosenberg et al. (29) found that low-frequency dTMS did not have a statistically significant effect on auditory hallucinations or other psychopathology in schizophrenia.

A systematic review (32) examined the efficacy and safety of dTMS in schizophrenia based on one RCT (30) and a single-arm perspective study (33), with obvious heterogeneity in methodology between studies; furthermore, this systematic review did not incorporate a recent double-blind RCT (31) on the efficacy and safety of dTMS in schizophrenia. To understand the current literature on the role of dTMS in schizophrenia and provide a more comprehensive and robust basis for clinical application, the present systematic review included three RCTs that evaluated the therapeutic efficacy and safety of dTMS in schizophrenia.

## Methods

### Search strategy and selection criteria

Three researchers, YM, XJL and CJD, independently conducted a literature search in four international (PubMed, EMBASE, PsycINFO, and Cochrane Library) and two Chinese (WanFang and Chinese Journal Net) databases from their inception to July 6, 2023. The search terms were as follows: (“deep transcranial magnetic stimulation” OR “deep repetitive transcranial magnetic stimulation” OR deep rTMS OR deep TMS OR dTMS OR H-coil) AND (schizophrenia [MeSH] OR schizophrenic disorder OR disorder, schizophrenic OR schizophrenic disorders OR schizophrenia OR dementia praecox) AND (random* OR sham OR placebo OR control). Furthermore, the reference lists of eligible articles (29–31) and a systematic review (32) were screened for additional studies.

In accordance with the PRISMA guidelines, the inclusion criteria of this systematic review were based on the PICOS acronym (34). *P*articipants: adults diagnosed with schizophrenia and schizoaffective disorder according to international criteria. *I*ntervention versus *C*omparison: active dTMS plus antipsychotics or no antipsychotics versus sham dTMS plus antipsychotics or no antipsychotics. *O*utcomes: the primary outcome was the change in total psychopathology at post-dTMS, measured using standardized instruments such as the Positive and Negative Syndrome Scale (PANSS) (35) or the Brief Psychiatric Rating Scale (BPRS) (36). Secondary outcomes included positive, negative, and general psychopathology scores of the PANSS, BPRS, the Scale for the Assessment of Positive Symptoms (SAPS) or the Scale for the Assessment of Negative Symptoms (SANS), auditory hallucinations measured by the Auditory Hallucination Rating Scale (AHRS) (37), neurocognitive function, discontinuation due to any reason, and adverse events. ***S*** tudy: published RCTs of dTMS for schizophrenia. Studies on active dTMS versus other forms of NIBS, review articles, and case reports/series were excluded.

### Data extraction

Data extraction from each included RCT was carried out independently by the same three researchers. A standardized form was used for data extraction that included authorship, publication year, study design, dTMS protocol, and primary and secondary outcomes. When additional information was required, the study author(s) were contacted to provide missing data.

### Study quality assessment

The quality of the RCTs was evaluated independently by the same three researchers using the Jadad scale (38) and the Cochrane risk of bias (39). RCTs with a Jadad score of ≥3 were classified as of “high quality” (40).

## Results

### Literature search

**Figure 1:**
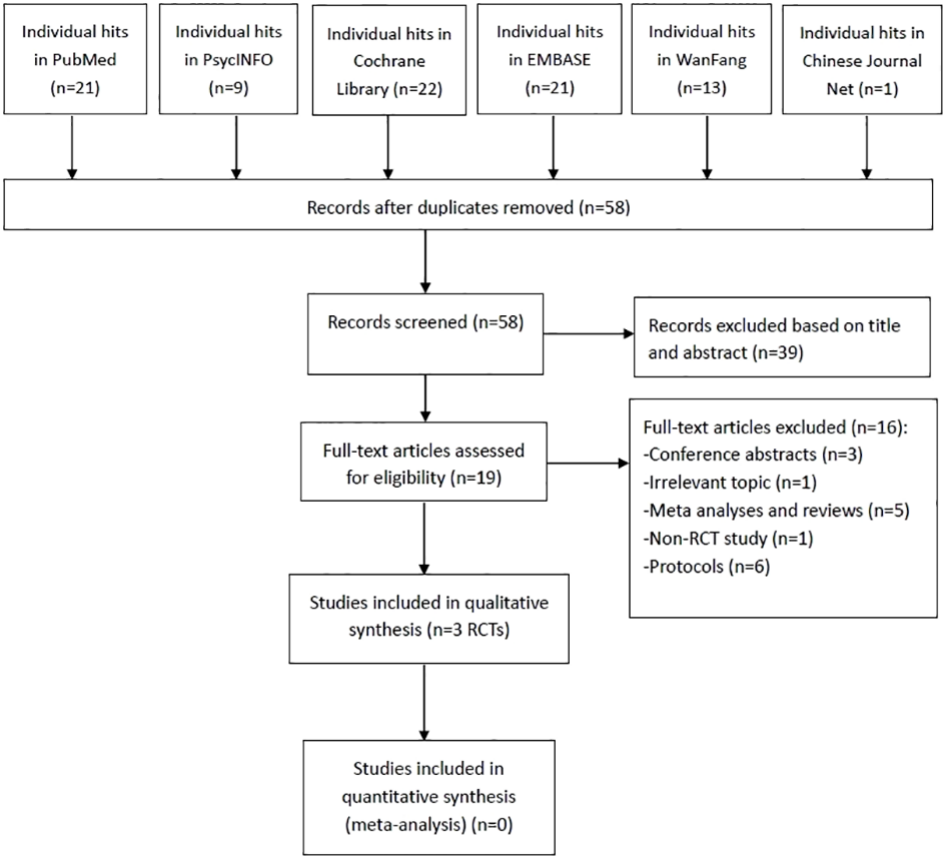
PRISMA flow diagram. PRISMA, Preferred Reporting Items for Systematic Reviews and Meta-analyses; RCTs, randomized controlled trials.

As shown in **Figure 1**, 87 studies were identified. After screening the titles, abstracts, and full texts independently by the same three researchers, three RCTs met the inclusion criteria (29–31). Due to the insufficient data, a meta-analysis could not be conducted.

### Study characteristics

**Table 1** shows the participants’ characteristics and dTMS parameters across the three RCTs (n = 80). These studies, published between 2012 and 2022, compared active dTMS (n = 39) with sham tACS (n = 29) in patients with schizophrenia or schizoaffective disorder. Two RCTs (2/3, 66.7%) were conducted in Israel, and one (1/3, 33.3%) in the USA (**Table 1**). The weighted mean age of the participants was 39.6 years, with 73.5% (range: 70.0%–88.3%) being male. Regarding treatment target, Moeller et al. (31) targeted bilateral insular and prefrontal cortices, while Rabany et al.’ (30) focused on the left dorsolateral prefrontal cortex, and Rosenberg et al.’ (29) aimed at the left temporoparietal cortex. The stimulation intensity and frequency in the protocols were different between studies, with the stimulation intensity ranging from 100% to 120%, and the frequency ranging from 1, 10 and 20 Hz (**Table 1**).

**Table 1:** Summary of the characteristics of the included studies.

Study (country)	Sample size (n)^a^	Design: -Blinding -Setting (%)	Participants: -Diagnosis -Diagnostic criteria -Illness duration (yrs)	Mean age (yrs) (range)	Gender: male (%)	dTMS treatment duration -Study duration	Intervention versus control groups; number of patients (n)	-Target site -Intensity -Frequency	-Coil type -Total session (n) -Trains/per session (n)	-Stimulus time/per session -Train length -Intertrain interval	Jadad score
Moeller et al., 2022 (31) (USA)	32^b^	-DB -NR	-SCZ/SzA -DSM-5 -NR	48.8 (NR)	14 (70.0)	-3 w -3 w	1. Active dTMS+APs or no APs; n=10 2. Sham dTMS+APs; n=10	-Bilateral insular and prefrontal cortices -100%-120% MT -10 Hz	-H4 -15 -60	-20 min -3 s -15 s	4
Rabany et al., 2014 (30) (Israel)	30	-DB -NR	-SCZ/SzA -ICD-10 -11.4	34.0 (NR)	21 (70.0)	-4 w -8 w	1. Active dTMS+APs; n=20 2. Sham dTMS+APs; n=10	-Left DLPFC -120% MT -20 Hz	-H1 -20 -42	-NR -2 s -20 s	3
Rosenberg et al., 2012 (29) (Israel)	18	-DB -Inpatients (5.6) and outpatients (94.4)	-SCZ -DSM-IV-TR -14.9	38.8 (19–63)	15 (88.3)	-10 d -10 d	1. Active dTMS+APs; n=9 2. Sham dTMS+APs; n=9	-Left temporoparietal cortex -110% MT -1 Hz	-H1 -10 -NR	-10 min -NR -NR	4

a Data were extracted based on random assignment.

b A total of 32 patients were randomized and assigned to either active or sham treatment. The final analysis included 20 patients who completed the 3-week dTMS treatment, as 12 patients were withdrawn.

APs, antipsychotics; d, days; DB, double blind; DLPFC, dorsolateral prefrontal cortex; DSM-IV-TR, Diagnostic and Statistical Manual of Mental Disorders, fourth edition text revision; DSM-5, Diagnostic and Statistical Manual of Mental Disorders 5th edition; dTMS, deep transcranial magnetic stimulation; H1, H1 coil; H4, H4 coil; ICD-10, International Classification of Diseases, 10th edition; MT, motor threshold; min, minutes; NR, not reported; s, seconds; SCZ, schizophrenia; SzA, schizoaffective disorder; TRD, treatment-resistant depression; w, weeks; yrs, years.

### Quality assessment

**Figure 2:**
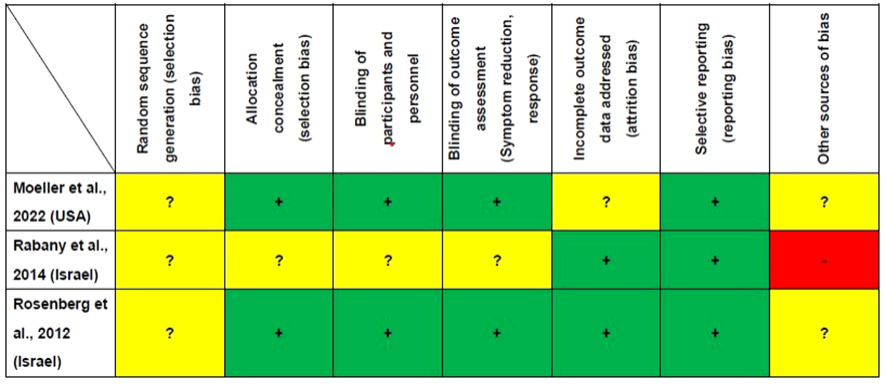
Cochrane risk of bias. +: Low risk of bias, -: High risk of bias,?: Unclear risk of bias.

**Figure 2** presents the study quality assessment of the three RCTs. Two RCTs (2/3, 66.7%) were deemed ‘low risk’ for allocation concealment, blinding of participants and personnel and blinding of outcome assessment (29, 31). Two RCTs (2/3, 66.7%) were classified as ‘low risk’ for incomplete outcome data (29, 30). All the three RCTs were rated as ‘low risk’ for selective reporting (29–31). The mean Jadad score was 3.7 (range: 3–4), indicating that all the studies were of high quality (Jadad score ≥ 3) (**Table 1**).

### Psychopathology

**Table 2** displays the findings consistently demonstrating that active dTMS yielded similar improvements in total psychopathology, positive symptoms, negative symptoms, and auditory hallucinations compared to the sham treatment. Rabany et al.’s study (30) reported a significant reduction in negative symptoms in the dTMS group, although no significant differences were observed between the two groups.

**Table 2:** dTMSfor schizophrenia: clinical efficacy.

Study	Clinical symptoms	Findings
Moeller et al., 2022 (31) (USA)	PANSS^a^	-No significant differences were found regarding positive symptoms, negative symptoms and general psychopathology symptoms between active dTMS group and sham group. -Response (%): NR. -Remission (%): NR.
Rabany et al., 2014 (30) (Israel)	PANSS^b^ and SANS	-No significant group differences were found regarding total psychopathology and negative symptoms. -Response^c^ (%): active dTMS group vs. sham group = 62.5 vs.33.3. -Remission (%): NR.
Rosenberg et al., 2012 (29) (Israel)	AHRS, SANS, and SAPS	-No significant differences were found regarding positive symptoms, negative symptoms and auditory hallucinations between active dTMS group and sham group. -Response (%): NR. -Remission (%): NR.

a PANSS (positive symptoms, negative symptoms and general psychopathology symptoms).

b PANSS (total psychopathology and negative symptoms).

c Defined as a change from baseline SANS score by at least 20%. AHRS, Auditory Hallucinations Rating Scale; dTMS, deep transcranial magnetic stimulation; NR, not reported; PANSS, Positive and Negative Syndrome Scale; SANS, Scale for the Assessment of Negative Symptoms; SAPS, Scale for the Assessment of Positive Symptoms.

### Neurocognitive function

Only one study examined the impact of dTMS on neurocognitive function (30) (**Table 3**): active dTMS improved only one Stockings of Cambridge measure (subsequent times for five move problems) at week 4 (P < 0.05) using the Cambridge Neuropsychological Test Automated Battery (CANTAB). However, no significant effects were observed on psychomotor speed, visuospatial memory, and sustained attention measured by the CANTAB.

**Table 3:** Active versus sham dTMS for patients with schizophrenia: neurocognitive function.

Study	Neurocognitive function	Findings
Moeller et al., 2022 (31) (USA)	None	None
Rabany et al., 2014 (30) (Israel)	CANTAB	No differences were found between the groups in score changes in any of the neurocognitive tests from baseline to any of the time points, apart from one Stockings of Cambridge measure (i.e., subsequent times for five move problems) at week 4.
Rosenberg et al., 2012 (29) (Israel)	None	None

CANTAB, Cambridge Neuropsychological Test Automated Battery; dTMS, deep transcranial magnetic stimulation.

### Discontinuation rate and adverse events

**Table 4** presents the discontinuation rates reported in the three RCTs, ranging from 16.7% to 44.4%. Adverse events were assessed in only one RCT (31). Adverse events associated with dTMS included tingling/twitching (3/10, 30.0%), head/facial discomfort (3/10, 30.0%), back pain (2/10, 20.0%), and accidental falls (1/10, 10.0%).

**Table 4:** dTMS for schizophrenia: discontinuation rate and adverse effects.

Study	Discontinuation rate (n, %)	Active group (n, %)	Sham group (n, %)
Moeller et al., 2022 (31) (USA)	12 (37.5)	NR	NR
Rabany et al., 2014 (30) (Israel)	5 (16.7)	NR	NR
Rosenberg et al., 2012 (29) (Israel)	8 (44.4)	4 (44.4)	4(44.4)
Study	Adverse effects	Active group (n, %)	Sham group (n, %)
Moeller et al., 2022 (31) (USA)	Accidental fall	1 (10.0)	0 (0)
	Back pain	2 (20.0)	0 (0)
	Head/facial discomfort	3 (30.0)	0 (0)
	Neck/chest discomfort	0 (0)	1 (10.0)
	A feeling of overstimulation	2 (20.0)	0 (0)
	Tingling/twitching	3 (30.0)	0 (0)
Rabany et al., 2014 (30) (Israel)	NR	NR	NR
Rosenberg et al., 2012 (29) (Israel)	NR	NR	NR

dTMS, deep transcranial magnetic stimulation; NA, not applicable; NR, not reported; NS, not significant.

## Discussion

This systematic review included three RCTs involving 80 patients with schizophrenia. The main findings indicate that dTMS was not effective in reducing total psychopathology, positive psychopathology, negative psychopathology, or auditory hallucinations in schizophrenia, measured by the PANSS, SAPS, SANS, and AHRS, respectively. One RCT reported a relatively high rate of discontinuation of dTMS for various reasons (29). The most commonly reported adverse effects, assessed in only one RCT (31), included tingling/twitching, head/facial discomfort, and back pain. While dTMS does not appear to be a promising adjunctive therapy for schizophrenia, it shows potential for improving executive functions, suggesting the need for further studies with larger sample sizes to confirm this finding (30).

This systematic review indicates a lack of evidence to support the antipsychotic effects of dTMS in schizophrenia, particularly for refractory auditory hallucinations that may involve multiple brain regions. Previous meta-analyses have shown a negative correlation between the severity of auditory hallucinations and the volume of grey matter in the left insula and right superior temporal gyrus (41, 42). Additionally, a meta-analysis of diffusion-tensor-imaging studies comparing the brain network structure between schizophrenia patients with auditory hallucinations and healthy controls has identified defects in the left arcuate tract in the former group (43). Stimulation of the left temporoparietal cortex alone may not be sufficient, as inadequate activity in the prefrontal cortex and anterior cingulate cortex has also been linked to negative symptoms in schizophrenia (44, 45). High-frequency stimulation may improve activity in these regions, alleviating negative symptoms. However, a meta-analysis revealed that there is insufficient evidence to support or refute the use of TMS for treating psychotic symptoms in schizophrenia (11). This aligns with the findings of the present systematic review, which suggests that inadequate or inappropriate treatment parameters may contribute to the lack of efficacy. A network meta-analysis found that stimulation of the left dorsolateral prefrontal cortex resulted in significant improvements in psychotic symptoms (15). Only one RCT included in this review targeted the left dorsolateral prefrontal cortex for stimulation and reported a significant reduction in negative symptoms in the dTMS group (30). Considering the potential impact of dTMS on depression following schizophrenia, further investigation is warranted, given that a systematic review reported the efficacy of high-frequency dTMS in reducing depressive symptoms in major depression (23).

Neurocognitive function was assessed in only one RCT included in this systematic review (30), which did not find significant differences between the groups in the changes of neurocognitive test scores measured by the CANTAB, except for one executive function test, the Stocks of Cambridge. While dTMS can target deep regions, such as the anterior cingulate cortex (ACC) (46), which are associated with neurocognitive function (47), the results of dTMS on neurocognitive function in various populations have been inconsistent. Some studies suggest that dTMS may improve neurocognitive function in patients with depression (48, 49), while others have found no significant changes (50, 51). Similar results have been observed in studies involving healthy volunteers (52–55). The variability in results may be attributed to the lack of sensitivity of the assessment tools. Further research is needed to investigate the effects of dTMS on neurocognitive function using standardized and highly sensitive tools such as the MATRICS Consensus Cognitive Battery (56) or the Repeatable Battery for the Assessment of Neuropsychological Status (57).

The discontinuation rates for any reason and the occurrence of adverse events ranged from 16.7% to 44.4% in this systematic review, with only one study providing detailed numbers for discontinuation in each group (29). Although the discontinuation rate was high, there was no significant difference between the dTMS and sham groups. Adverse effects reported in one study (31) included tingling/twitching, head/facial discomfort, and back pain. These adverse effects were generally mild and transient.

This systematic review has the following limitations. First, it is worth noting that the sample sizes (n = 80) of the included studies were relatively small, which limits the generalizability of the findings and their ability to detect significant differences. Second, the three studies were too heterogeneous in their methods (e.g., the stimulation frequency of dTMS ranging from 1, 10 and 20 Hz, respectively) to allow for reliable and pertinent results. Third, the stimulation parameters of RCTs included in this systematic review were variable, therefore, the optimal stimulation parameters, such as stimulation time per session and train length, for the dTMS protocol need further exploration. Fourth, only one of the three included RCTs reported the neurocognitive effects of dTMS on schizophrenia patients. Further research is warranted to evaluate the neurocognitive effects of dTMS in patients with schizophrenia.

In conclusion, this systematic review found that dTMS does not appear to be effective in reducing psychopathology, including positive and negative symptoms, and auditory hallucinations in schizophrenia. This may call for further research on this technique and the development of protocols. Further research with larger, well-designed trials is needed to evaluate the efficacy and safety of dTMS in schizophrenia, particularly targeting specific brain regions and neurocognitive domains. Additionally, standardized and sensitive assessment tools should be used to evaluate the neurocognitive effects of dTMS in this population.

## Data availability statement

The raw data supporting the conclusions of this article will be made available by the authors, without undue reservation.

## Author contributions

YM: Writing – original draft, Data curation. Z-MS: Writing – original draft, Investigation. X-HY: Writing – review & editing, Writing – original draft, Methodology, Conceptualization. X-JL: Writing – review & editing, Resources, Methodology. C-JD: Writing – review & editing, Investigation, Data curation. X-BH: Writing – review & editing, Supervision, Project administration. X-LT: Writing – review & editing, Resources, Investigation. SP: Writing – review & editing, Supervision, Resources. GU: Writing – review & editing, Validation, Supervision. Y-TX: Writing – review & editing, Supervision. WZ: Writing – review & editing, Validation, Supervision.
